# 红细胞分布宽度在骨髓增生异常综合征患者中的预后价值

**DOI:** 10.3760/cma.j.issn.0253-2727.2022.08.013

**Published:** 2022-08

**Authors:** 莹 陈, 婷婷 牛, 淙 石, 多兵 邹, 桂芳 欧阳, 启田 牧, 洁 金

**Affiliations:** 1 浙江大学医学院附属第一医院血液科，浙江省血液肿瘤（诊治）重点实验室，杭州 310000 Department of Hematology, Institute of Hematology, The First Affiliated Hospital, Zhejiang University School of Medicine, Hangzhou 310000, China; 2 宁波市第一医院干细胞移植实验室，宁波 315010 Laboratory of Stem Cell Transplantation, Ningbo First Hospital, Ningbo 315010, China; 3 宁波市第一医院血液科，宁波 315010 Department of Hematology, Ningbo First Hospital, Ningbo 315010, China

骨髓增生异常综合征（MDS）是一种起源于造血干细胞的克隆性疾病，其特征为髓系细胞发育异常、分化障碍和外周血细胞减少等，并有向急性髓系白血病（AML）进展的风险[1]。由于遗传改变存在高度异质性，MDS患者预后有很大差异。临床上常用的MDS预后评估系统主要有修订版国际预后评分系统（IPSS-R）[2]和WHO预后评分系统（WPSS）[3]。近些年，染色体单体核型、一些基因突变等指标被报道是独立于这些评分系统外的预后不良因素[4]–[6]，提示现有的风险评估系统仍有待进一步完善。

红细胞分布宽度（RDW）是能反映红细胞体积异质性的指标，可用来区分不同类型的贫血。以往多项研究表明，依据RDW水平可辅助诊断MDS。Rauw等[7]研究发现，RDW对不明原因血细胞减少症患者进行MDS诊断具有独立预测价值。赵丽等[8]也报道平均红细胞体积（MCV）/RDW结合网织红细胞参数可作为再生障碍性贫血、巨幼红细胞贫血、溶血性贫血及MDS的鉴别指标。然而，对于RDW在MDS患者中的预后价值在国内尚未见相关报道。本研究我们探索RDW在初诊MDS患者中的预后价值。

## 病例与方法

1. 病例：回顾性分析2009年6月至2018年12月在宁波市第一医院血液科就诊的240例初诊MDS患者临床和实验室资料，并参照WHO（2016）标准[3]进行诊断分型，本研究通过宁波市第一医院伦理委员会审查批准（批件号：2021RS080）。

2. 二代测序（NGS）检测基因突变：提取MDS患者骨髓细胞，抽提基因组DNA，采用Ampliseq多重PCR扩增并构建样本文库，在Ion proton平台上进行高通量测序，测序后参考COSMIC数据库进行生物信息学分析，确定致病性基因突变位点，测序深度为2000×，等位基因突变频率（VAF）>1％认为是基因突变。由武汉康圣达医学检验所完成NGS检测。检测位点包括NRAS、DNMT3A、SF3B1、IDH1、TET2、EZH2、JAK2、CBL、ETV6、IDH2、TP53、SRSF2、ASXL1、RUNX1共14种常见MDS相关基因突变。

3. 治疗方案：240例MDS患者中，共有35例患者接受去甲基化治疗，36例接受化疗，160例仅接受对症治疗，其余9例患者治疗方案未知。去甲基化方案为地西他滨（10 mg·m^−2^·d^−1^，第1～7天）或阿扎胞苷（75 mg·m^−2^·d^−1^，第1～7天）；化疗方案为小剂量阿糖胞苷（10 mg/m^2^每12 h 1次，第1～14天）联合阿克拉霉素或高三尖杉酯碱或去甲氧柔红霉素。

4. 疗效评估及随访：通过查阅住院病历、门诊病历或电话进行随访。随访截至2019年12月24日，中位随访时间为20（0.3～125）个月。主要观测终点为总生存（OS）时间及无白血病生存（LFS）时间。OS时间指从确诊日期起至患者因任何原因死亡或末次随访时间；LFS时间指确诊日期至患者转化为白血病或死亡的时间[9]。

5. 统计学处理：应用R语言中的survival包surv_pvalue函数获得RDW截断值。使用SPSS 26.0软件进行分析。连续变量组间比较采用Mann-Whitney *U*检验，分类变量组间比较采用*χ*^2^检验或Fisher确切概率法，采用Kaplan-Meier法绘制生存曲线，Log-rank检验进行组间比较，采用Cox回归模型进行单因素与多因素分析，*P*<0.05为差异有统计学意义。

## 结果

1. 患者一般情况：240例MDS患者中，男133例，女107例，中位年龄62（16～90）岁。200例患者可进行IPSS-R预后分组，其中极低危组14例，低危组46例，中危组67例，高危组36例，极高危组37例。本组病例中，初诊时RDW中位数为16.0％（10.9％～34.9％），cut-off为16.6％，RDW≤16.6％为RDW较低组，共138例患者；RDW>16.6％为RDW较高组，共102例患者。与RDW较低组相比，RDW较高组具有更高年龄、环形铁粒幼红细胞比例、乳酸脱氢酶水平和血清铁蛋白水平，更低血红蛋白水平，在WHO分型各亚型分布中差异有统计学意义（*P*值均<0.05）。而性别、白细胞计数、血小板计数、骨髓原始细胞比例、网织红细胞比值、骨髓红系比例、IPSS-R预后分组、β_2_微球蛋白水平差异均无统计学意义（表1）。

**表1:** 240例骨髓增生异常综合征（MDS）患者总体及不同红细胞分布宽度（RDW）分组的临床与实验室资料

临床资料	总体（240例）	RDW较低组（138例）	RDW较高组（102例）	*P*值
性别［例（％）］				0.793
男	133（55.4）	75（54.3）	58（56.9）	
女	107（44.6）	63（45.7）	44（43.1）	
年龄［岁，*M*（范围）］	62（16～90）	59（16～85）	66（18～90）	0.007
WBC［×10^9^/L，*M*（范围）］	2.8（0.5～15.4）	2.7（0.5～9.5）	2.9（0.7～15.4）	0.717
HGB［g/L，*M*（范围）］	76（22～142）	91（22～142）	66（23～123）	<0.001
PLT［×10^9^/L，*M*（范围）］	52（2～434）	53（2～332）	49（2～434）	0.676
骨髓原始细胞比例［％，*M*（范围）］	4.0（0～19.5）	4.5（0～19.5）	3.0（0～19.5）	0.339
网织红细胞比值^a^［％，*M*（范围）］	1.6（0.1～11.2）	1.6（0.1～5.9）	1.7（0.1～11.2）	0.422
环形铁粒幼红细胞比例［例（％）］				0.045
≥15％	13（5.4）	4（2.9）	9（8.8）	
<15％	227（94.6）	134（97.1）	93（91.2）	
骨髓红系比例［％，*M*（范围）］	31.0（0～78.5）	28.5（3.0～74.5）	33.7（0～78.5）	0.061
IPSS-R预后分组^b^［例（％）］				0.093
极低危组	14（7.0）	12（10.1）	2（2.5）	
低危组	46（23.0）	30（25.2）	16（19.8）	
中危组	67（33.5）	35（29.4）	32（39.5）	
高危组	36（18.0）	23（19.3）	13（16.0）	
极高危组	37（18.5）	19（16.0）	18（22.2）	
β_2_微球蛋白^c^［mg/L，*M*（范围）］	1.93（0.04～12.61）	1.83（0.04～8.75）	2.18（0.40～12.61）	0.136
LDH ^c^［U/L，*M*（范围）］	207（54～1083）	202（54～789）	212（94～1083）	0.030
血清铁蛋白^d^［µg/L，*M*（范围）］	310（5～2612）	305（5～1500）	310（5～2612）	0.035
WHO分型［例（％）］				0.012
MDS-SLD	26（10.8）	21（15.2）	5（4.9）	
MDS-MLD	73（30.4）	40（29.0）	33（32.4）	
MDS-RS-SLD	3（1.3）	0（0.0）	3（2.9）	
MDS-RS-MLD	13（5.4）	4（2.9）	9（8.8）	
MDS-5q−	4（1.7）	2（1.5）	2（2.0）	
MDS-EB-1	47（19.6）	29（21.0）	18（17.7）	
MDS-EB-2	61（25.4）	37（26.8）	24（23.5）	
MDS-U	13（5.4）	5（3.6）	8（7.8）	

注：IPSS-R：修订版国际预后积分系统；MDS-SLD：MDS伴单系病态造血；MDS-MLD：MDS伴多系病态造血；MDS-RS-SLD：MDS伴环状铁粒幼红细胞和单系病态造血；MDS-RS-MLD：MDS伴环状铁粒幼红细胞和多系病态造血；MDS-5q−：MDS伴有单纯5q−；MDS-EB1：MDS伴原始细胞增多-1型；MDS-EB2：MDS伴原始细胞增多-2型；MDS-U：MDS未分型。a：144例患者有初诊时网织红细胞计数结果；b：200例患者可进行IPSS-R预后分组；c：220例患者有初诊时β_2_微球蛋白和LDH结果；d：190例患者有初诊时血清铁蛋白结果

2. RDW升高与基因突变种类分析：在240例MDS患者中，68例患者进行14种MDS相关基因突变检测，中位突变1（0～8）个，40例（58.82％）患者中检测到至少1个基因突变。ASXL1基因突变频率最高，占19.12％；未检出IDH1基因突变。RDW较低组和RDW较高组各基因突变率差异均无统计学意义（*P*值均<0.05）（表2）。

**表2:** 68例骨髓增生异常综合征（MDS）患者不同红细胞分布宽度（RDW）分组中14种基因突变率比较

基因	RDW较低组（48例）	RDW较高组（20例）	*P*值
SF3B1	3（6.25）	3（0.15）	0.246
P53	8（16.67）	1（0.05）	0.196
TET2	9（18.75）	2（0.10）	0.372
RUNX1	8（16.67）	4（0.20）	0.743
ASXL1	11（22.92）	2（0.10）	0.217
DNMT3A	3（6.25）	1（0.05）	0.842
NRAS	2（4.17）	0（0.00）	0.354
IDH1	0（0.00）	0（0.00）	1.000
EZH2	4（8.33）	1（0.05）	0.631
CBL	1（2.08）	1（0.05）	0.517
SRSF2	7（14.58）	0（0.00）	0.071
IDH2	2（4.17）	1（0.05）	0.879
ETV6	3（6.25）	0（0.00）	0.253

3. 生存分析：在240例MDS患者中，RDW较低组共有21例患者进展为急性白血病，64例患者死亡；RDW较高组共有9例患者进展为急性白血病，62例患者死亡。RDW较高组中位OS时间分别为19.7（95％ *CI* 12.2～27.2）个月，明显短于RDW减低组的45.6（95％ *CI* 29.2～62.0）个月（*P*＝0.009）（图1 A）。RDW较高组中位LFS时间为19.4（95％ *CI* 10.4～28.3）个月，有短于RDW较低组的趋势［42.0（95％ *CI* 12.9～71.1）个月，*P*＝0.075］（图1 B）。

**图1:**
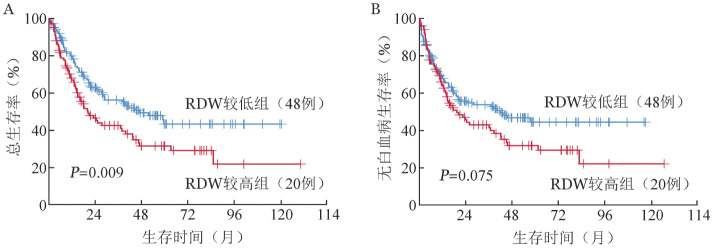
骨髓增生异常综合征（MDS）患者不同红细胞分布宽度（RDW）水平分组的总生存（A）、无白血病生存（B）曲线

4. 预后影响因素分析：对240例MDS患者进行单因素分析，结果显示年龄、性别、IPSS-R分组、血清铁蛋白、β_2_微球蛋白、LDH及RDW是OS和LFS的影响因素（*P*值均<0.05），而去甲基化治疗仅对LFS有影响（*P*＝0.001）。将单因素分析*P*<0.1的变量纳入多因素分析，结果显示年龄、性别、IPSS-R分组、去甲基化治疗是OS的独立影响因素，年龄、性别、去甲基化治疗是LFS的独立影响因素。而RDW水平在多因素分析中未显示出统计学意义（表3）。

**表3:** 影响骨髓增生异常综合征（MDS）患者总生存（OS）与无白血病生存（LFS）的单因素和多因素分析

影响因素	OS				LFS			
	单因素分析		多因素分析		单因素分析		多因素分析	
	*OR*（95％*CI*）	*P*值	*OR*（95％*CI*）	*P*值	*OR*（95％*CI*）	*P*值	*OR*（95％*CI*）	*P*值
RDW>16.6％	1.595（1.122~2.267）	0.009	0.944（0.568~1.569）	0.824	1.369（0.967~1.938）	0.076	0.821（0.498~1.353）	0.438
年龄≥60岁	2.518（1.721~3.682）	<0.001	3.312（1.764~5.216）	<0.001	2.184（1.508~3.165）	<0.001	2.942（1.604~5.396）	<0.001
男性	0.518（0.356~0.752）	0.001	0.407（0.228~0.727）	0.002	0.538（0.373~0.777）	0.001	0.475（0.272~0.830）	0.009
IPSS-R较高危组	1.426（1.191~1.706）	<0.001	1.357（1.065~1.730）	0.014	1.434（1.199~1.715）	<0.001	1.244（0.971~1.593）	0.084
未接受去甲基化治疗	1.261（0.994~1.599）	0.056	1.633（1.162~2.295）	0.005	1.485（1.185~1.860）	0.001	1.791（1.310~2.450）	<0.001
血清铁蛋白>338 µg/L	1.845（1.233~2.759）	0.003	0.970（0.576~1.636）	0.910	1.942（1.303~2.893）	0.001	1.113（0.659~1.880）	0.689
β_2_微球蛋白>1.3 mg/L	2.251（1.467~3.452）	<0.001	1.448（0.774~2.710）	0.247	1.890（1.228~2.909）	0.004	1.207（0.640~2.275）	0.562
LDH>250 U/L	2.338（1.611~3.391）	<0.001	1.409（0.835~2.377）	0.199	2.333（1.614~3.372）	<0.001	1.246（0.732~2.121）	0.418

注：RDW：红细胞分布宽度

## 讨论

本研究通过回顾性研究分析2009年到2018年初诊的240例MDS患者数据，探讨RDW水平对患者预后的影响。Baba等[10]研究发现RDW cut-off为15％时，RDW水平升高与MDS-RA患者不良预后有关。此外，也有文献报道将15.5％作为cut-off在MDS患者中进行研究分析[11]。本研究中RDW的cut-off为16.6％，与上述文献相近。本研究结果显示，RDW较高组患者具有更低血红蛋白（*P*<0.001）和更高血清铁蛋白（*P*＝0.035）水平。RDW是全血细胞分析中反映红细胞体积的异质性指标，红细胞体积异质性增大提示血红蛋白合成异常和红细胞生成缺陷[12]。最近研究表明，RDW与各种癌症的发生或预后密切相关[13]–[15]，虽然在肿瘤中RDW形成的机制尚未阐明，但大多数研究发现RDW升高可能与肿瘤介导的炎症微环境相关[16]。炎症微环境可能对体内铁代谢造成损伤，抑制促红细胞生成素的产生，使得大量未成熟红细胞由骨髓进入外周血循环，外周血中的红细胞大小不一致，最终导致RDW升高[17]–[18]。血红蛋白作为携氧介质，是红细胞的重要组成部分。Wang等[19]研究发现氧化应激反应是导致红细胞大小发生变化的重要因素，游离的活性氧会损害蛋白质、脂质及DNA，从而降低血红蛋白水平。

本研究中部分患者进行14种基因突变检测，58.82％的患者伴有至少1个基因突变，各基因突变率与文献[20]–[21]报道接近，但基因突变在不同RDW水平中无显著差异。Shi等[22]发现，SF3B1基因突变在RDW较低组和较高组中存在显著差异。SF3B1基因突变常见于含有环形铁粒幼细胞的MDS中，与RS亚型密切相关。有研究报道显示，用SF3B1基因最常见的突变体Sf3b1（K700E）构建的小鼠模型，会出现大红细胞性贫血、红系发育不良和造血干细胞扩增[23]–[24]。本研究表明，RDW较高组中环形铁粒幼细胞比例明显高于RDW较低组，而SF3B1基因突变率在两组间无统计学差异，这可能与进行基因检测病例少数有关。除SF3B1基因突变率外，两组之间其余基因突变率比较与该研究相符。

我们对MDS预后进行多因素分析，结果显示IPSS-R积分在OS中统计学差异最为明显，IPSS-R积分越高，患者的OS越短，这一结果与其他文献一致[9],[25]。此外，本研究显示治疗方式是OS及LFS的独立预后因素。DNA甲基化是基因转录的重要调节因子，在MDS的发病机制中起到关键作用[26]。阿扎胞苷和地西他滨作为最常见的去甲基化药，已在MDS中广泛使用。与单独接受支持性治疗的患者相比，接受地西他滨的患者具有更长的LFS[27]。同样，在高风险MDS患者中，阿扎胞苷不仅能改善患者的生活质量、显著延长患者转白时间，还能改善红细胞输注需求和感染率[28]。通过Kaplan-Meier分析，我们发现RDW较高组OS明显差于RDW较低组，而两组患者的LFS无明显差异。然而，进一步进行多因素分析，RDW升高对两组患者OS的影响无统计学差异。Baba等[10]研究发现，在MDS-RA亚型中，高RDW水平患者具有更短OS时间，但由于病例数有限，作者并未进行多因素分析。Shi等[22]报道RDW升高在原始细胞数<5％的MDS患者中是独立预后因素。上述结果说明原始细胞比例可能对RDW作为独立预后指标的干扰性较大。尽管本研究MDS病例数有240例，仍需要将来多中心扩大样本进一步验证RDW的独立预后价值。

得益于各种MDS评分系统的建立和不断完善，MDS患者预后评估的准确性有了很大提高。RDW为血常规中的一个指标，检测方式简单、快速且廉价，对于染色体核型分析失败的MDS患者，RDW可作为一个简单而易行预后评价的指标。RDW升高在MDS患者中提示不良预后，可作为IPSS-R和WPSS预后评分系统有益的补充。
